# Not like us: Exploring the cardiovascular consequences of ultramarathons

**DOI:** 10.1113/EP092003

**Published:** 2024-06-15

**Authors:** Matthew C. Babcock, Austin T. Robinson

**Affiliations:** ^1^ Division of Geriatric Medicine, School of Medicine University of Colorado Anschutz Medical Campus Aurora Colorado USA; ^2^ Department of Kinesiology, School of Public Health Indiana University Bloomington Indiana USA

**Keywords:** cardiovascular physiology, endothelial function, exercise science, flow mediated dilation, ultramarathon

Physical inactivity is a major modifiable risk factor for cardiovascular and other diseases. For example, taking more steps per day is associated with progressively lower risk of all‐cause mortality (Paluch et al., 2022). Exercise is a subset of physical activity that is planned and structured, with the objective of improving physical fitness, and involves higher‐intensity activity, such as running. There is a growing concern that ‘excessive’ exercise volume might reverse some of the cardiovascular benefits of physical activity, and observational studies indicate accelerated coronary artery calcification and atrial fibrillation in veteran endurance adults compared with moderately active individuals (Franklin et al., 2020). These findings challenge the dogma that more exercise is better and suggest a potential ‘J‐shaped’ relationship, whereby very high levels of exercise might increase cardiovascular risk. However, the relatively small number of people who reach these extraordinarily high levels of exercise make it difficult to determine whether there is a threshold at which exercise volume is ‘too much’.

Participation in ultramarathons (i.e., any running event longer than a marathon, or 42.2 km) is growing dramatically, with the number of races and participants in those races increasing exponentially over the past ∼30 years (Ronto, 2020). Although ultra‐endurance runners still represent a relatively small subset of the overall running community, the growing number of ultra‐endurance athletes results in a unique population for researchers to investigate extreme exercisers. In this issue of *Experimental Physiology*, Ranadive et al. (2024) leveraged this unique population to examine the acute effects of ‘extreme’ endurance exercise on peripheral micro‐ and macrovascular function and central haemodynamics before, during and after a 50 km ultramarathon. Additionally, they examined serial measures of circulating inflammatory markers to gain mechanistic insight into any changes observed. In contrast to previous findings of increased arterial stiffness within 60 min of finishing either a 120 or 195 km trail ultramarathon (Burr et al., 2012), this study found no change in macrovascular function assessed via brachial artery flow‐mediated dilatation (FMD) during, immediately after or 24 h after a 50 km ultramarathon, despite dramatic increases in circulating pro‐inflammatory cytokines. However, the authors observed a reduction in microvascular function assessed via reactive hyperaemia area under the curve immediately after the race. This is again in contrast to previous findings regarding arterial stiffness in small arteries, in which there was no change in estimates of microvascular arterial stiffness (Burr et al., 2012). The contrasting findings from previous research could be attributable to differences in exercise stimulus (50 km in the present study vs. 120 or 195 km in prior studies), differences in the effects of ultra‐endurance exercise on endothelial function versus arterial stiffness, or other factors, such as runner characteristics and environmental conditions.

It should be noted that 50 km is the shortest (popular) race distance beyond a marathon and is likely to be more similar to the marathon exercise stimulus than other distances included in the ‘ultramarathon’ category. Other popular ultramarathon distances include, but are not limited to, 50 miles (80 km), 100 km, 100 miles (161 km), and the growing popularity of 200 miles (∼325 km), which cover much larger increases in distance than the difference between a 42.2 km marathon and 50 km ultramarathon. Therefore, the marathon might provide a more appropriate comparison to a 50 km ultramarathon, rather than using other ultramarathons as a comparison. One previous study examined the acute effects of a marathon on arterial stiffness and found no difference in large artery arterial stiffness from pre‐ to post‐marathon (Vlachopoulos et al., 2010). This race distance is much more comparable to the 50 km in the study by Ranadive et al. (2024) (∼1.2× longer) than the much longer ultramarathons in the study by Burr et al. (2012) (∼3× and 4.5× longer), suggesting that differences in the findings by Burr et al. (2012) might be the result of the larger exercise stimulus.

A unique aspect of the race used in this study is that it covered five laps of a 10 km course, allowing the researchers to study macro‐ and microvascular function and central haemodynamics multiple times throughout the race. Furthermore, environmental conditions were relatively mild; average elevation was ∼120 m, and temperatures ranged from 4 to 8°C, in contrast to many races where temperatures can range from very hot (∼40–42°C at the Western States Endurance Run in the Eastern Sierra Mountains of California) to very cold (∼−5°C at Ultra‐Trail du Mont Blanc in the Alps of France, Italy and Switzerland). These mild temperatures and near‐sea‐level elevation minimize the influence of environmental conditions in this study and allow the authors to draw conclusions related to the effects of ‘extreme’ endurance exercise from this field study.

Although the study in this issue of *Experimental Physiology* does not seek to determine the long‐term effects of ultramarathon participation on cardiovascular health, the athletes exhibited an average brachial artery FMD of 6.4%. Based on published reference data, a sample of ∼40‐year‐olds at the 50th percentile of brachial artery FMD consisting of eight males and three females would present with an FMD of 6.1% (Holder et al., 2021). It is also well accepted that athletes typically have larger arterial diameters, owing to exercise adaptations (Green et al., 2013). Without scaling for the structural arterial enlargement, which impacts the FMD calculation, vasodilator capacity can appear artificially blunted in athletes compared with less active individuals (Green et al., 2013). Considering this information, the resting 6.4% FMD in the athletes suggests that the athletes in the present study exhibited healthy vascular function. Although the present study did not conduct gold‐standard measurements of cardiac function or arterial stiffness, these FMD findings suggest that 50 km ultramarathon training does not impair macrovascular function and partly challenge the hypothesized ‘J‐shaped’ relationship between exercise volume and cardiovascular health or at least suggest that the ‘J’ is shifted further to the right, towards even higher endurance exercise volume. As more studies of this nature are conducted, we will gain further insight into this hypothesized relationship between and the potential threshold at which exercise training might start to confer cardiovascular harm.

## AUTHOR CONTRIBUTIONS

Both authors have read and approved the final version of this manuscript and agree to be accountable for all aspects of the work in ensuring that questions related to the accuracy or integrity of any part of the work are appropriately investigated and resolved. All persons designated as authors qualify for authorship, and all those who qualify for authorship are listed.

## CONFLICT OF INTEREST

The authors declare no conflicts of interest.
